# Upper limb arthropathy in acromegaly: Radiological findings and functional impairment

**DOI:** 10.1007/s11102-026-01784-5

**Published:** 2026-11-13

**Authors:** Matheus Nejar Coan, Diego Paixão Côrtes Aguiar, Gabriela de Aguiar dos Santos, Lucas Elias Lise Simoneti, Julia Fernanda Semmelmann Pereira Lima, Miriam da Costa Oliveira

**Affiliations:** 1 Neuroendocrinology Center, Universidade Federal de Ciências da Saúde de Porto Alegre (UFCSPA), R. Sarmento Leite, 245 - Centro Histórico, Porto Alegre, RS 90050-170 Brazil; 2 Diagnostic Imaging Center, Santa Casa de Misericórdia de Porto Alegre, Porto Alegre, RS Brazil

**Keywords:** Acromegaly, Arthropathy, Osteoarthritis, Upper extremity,, Functional disability

## Abstract

**Purpose:**

Acromegaly is a rare disorder characterized by excessive growth hormone (GH) and insulin-like growth factor 1 (IGF-1) secretion, causing multiple systemic complications, including arthropathy, a major cause of functional disability. This study evaluated joint complaints and upper limb structural damage in patients with acromegaly.

**Methods:**

In this prospective study, patients with uncontrolled, medically controlled, or cured acromegaly underwent medical record review, assessment of joint complaints, QuickDASH evaluation, and shoulder and hand radiography.

**Results:**

48 patients with acromegaly (mean age, 51 ± 11.4 years) were included. Four tumors were microadenomas, and 42 were macroadenomas (including two giant adenomas), with invasive features in 20 cases. 46 patients underwent surgery, and 22 (45.8%) were receiving a somatostatin analog or pegvisomant at the last follow-up. In the specific questionnaire, 41 patients (85.4%) reported at least one joint complaint. QuickDASH showed upper limb functional limitations in 36 patients (75%): mild in 12, moderate in 14, and severe in 10. Radiographs were obtained in 27 patients and showed structural changes in 21 (77.7%), involving the hands in 70.3% and the shoulders in 59.3%. Glenohumeral osteoarthritis was diagnosed in 13 cases and acromioclavicular osteoarthritis in 10 cases. Joint complaints and biochemical disease control were not associated with employment status.

**Conclusion:**

Joint complaints and structural abnormalities are highly prevalent in acromegaly and may substantially reduce the quality of life for patients and their families.

## Introduction

Acromegaly, a disease caused by the hypersecretion of growth hormone (GH) and its mediator, insulin-like growth factor (IGF-1), is associated with increased morbidity and mortality [1]. Acromegaly comorbidities include acral and soft tissue overgrowth, osteoarticular complications, respiratory alterations, and hypertension, as well as metabolic abnormalities, such as insulin resistance, diabetes mellitus, malignancy, and cardiovascular disease [2]. The osteoarticular system may be affected in many forms, including fragility fractures such as vertebral fractures, kyphosis, osteoarthrosis, carpal tunnel syndrome (especially bilateral or relapsing), osteoporosis, arthropathy with articular pain, functional impairment, and swollen hands and feet [3, 4]. Joint manifestations are present at diagnosis in most patients [5]. Arthropathy is associated with pain and functional disability and has a significant impact on the patient’s daily activities [6]. Joint pain, stiffness, and functional impairment are closely associated with reduced QoL (HQ) [4, 6]. Additionally, arthropathy-related motor disability is associated with depression that independently contributes with similar strength to the impairment of QoL [7]. Even after biochemical control of acromegaly, residual sequelae may persist and significantly affect the patient’s life [7]. Progressive radiographic OA was observed in more than 70% of patients after 2.6 years of persistent remission [8].

Radiographic changes occur in up to 90% of patients with acromegaly [9]. The most commonly affected joints are those of the spinal column and lumbar region, with prevalences of 92% and 88%, respectively, followed by the distal and proximal interphalangeal joints and the first metacarpal joint [10]. Until recently, data on shoulder joints were scarce.

This study aimed to assess arthropathy in patients with acromegaly by evaluating spontaneous complaints and responses to specific questions about the joints and radiographic findings of the hands and shoulders, and correlating these with demographic variables. Additionally, the study aimed to evaluate the impact of joint injuries on daily life and to assess employability.

## Methodology

This cross-sectional observational study was conducted on a clinical cohort of 121 patients with acromegaly followed at a Neuroendocrinology Center, with diagnoses established on clinical and laboratory grounds. Certain data, such as spontaneously reported joint symptoms, were collected retrospectively, whereas questionnaires and radiological examinations were performed at the time of study inclusion. The recruitment period extended from March to November 2024, and patients were included consecutively after signing the informed consent form. The sample includes patients with uncontrolled, pharmacologically controlled, and cured acromegaly, as determined by sex- and age-adjusted IGF-1 values, at the last visit. Additionally, the disease control criteria that use IGF-1 threshold values up to 1.2 times the upper limit of normal (ULN) were also analyzed in the sample. Inclusion criteria were patients with more than 6 months of follow-up after pituitary surgery or more than 3 months after starting primary drug treatment. Exclusion criteria were a diagnosis of inflammatory rheumatic diseases. The medical records were reviewed for demographic, clinical, laboratory, and imaging data. Spontaneous reports of symptoms associated with arthropathies were collected from the records (this information was unavailable in five cases). At the time of inclusion in the study, patients answered specific questions about joint symptoms: pain and/or swelling in the joint, a “catching” sensation when bending or moving the joint, a crackling, grating, or popping sound and sensation that occurs when bending or moving the joint, an inability to fully extend or bend the joint, sensation of joint instability, stiffness, reduced range of motion. Functional disabilities were assessed using QuickDASH, a shortened version of the “Measures of adult shoulder function: Disabilities of the arm, shoulder, and hand questionnaire” in a validated Portuguese translation [11]. The same physician supervised completion of the self-administered QuickDASH. Employability was assessed using the “Occupation Status” questionnaire from the “Pesquisa Nacional por Amostra de Domicílios (PNAD Contínua)” [12]. This source considers an individual aged 60 or older to be elderly.

Radiographs of the hands and shoulders were ordered for all the patients included in the study. Due to operational difficulties, particularly those linked to patients’ social conditions. Only 27 patients returned within the study timeframe with the requested radiographic examinations. The radiological exams were performed according to the institutional radiology service protocols. The senior radiologist was blinded to the patient’s symptoms and biochemical disease status. For osteoarthritis classification, the modified Kellgren and Lawrence (KL score) system was used, as described in previous studies [13], interpreted by an experienced radiologist. The system uses 5 points (0 to 4) on a Likert scale and evaluates osteophytosis, joint space narrowing, sclerosis, and degenerative cysts to determine osteoarthritis severity for each joint evaluated. Radiographic osteoarthritis in a particular joint site is defined as a KL score of 2 or more. The carpometacarpal and thumb interphalangeal joints, as well as the sum of the metacarpophalangeal, proximal interphalangeal, and distal interphalangeal joints, are evaluated in the right and left hands; and, in the shoulder, the acromioclavicular and glenohumeral joints are assessed. The radiographic score for each patient was the average of the eight items, with individual scores.

The results for the qualitative variables were presented using absolute and relative frequencies, and for the quantitative variables using mean and standard deviation when symmetrical, and median, minimum, and maximum when asymmetrical. Normality was verified using the K-S test. The relationship between radiological evaluation, joint complaints, disabilities, and employability with clinical variables was assessed using Chi-square tests with adjusted standardized residuals, Mann-Whitney tests, Kruskal-Wallis tests with Dunn’s test for multiple comparisons, and Spearman’s correlation coefficient. After the original analyses, and taking into account the subgroup of patients with radiographic exams, we performed adjusted analyses correcting for multiple comparisons, applying the method of Benjamini-Hochberg to control the false discovery rate (FDR), considering the sets of associations related to each outcome of interest (radiological assessment, number of joint complaints, disability score, and employability) as families of tests. The significance level adopted was 0.05, and the analyses were performed using the SPSS statistical software (IBM SPSS Statistics for Windows, Version 25.0. Armonk, NY: IBM Corp.). The hospital’s Ethics and Research Committee approved the project under number 6.287.248, and all individuals signed an Informed Consent Form. No invited patient declined to participate in the study.

## Results

### Sample characterization

The sample included 48 patients with acromegaly, aged 30 to 74 years (51 ± 11.4 at diagnosis), of whom 52.1% were female. Regarding tumor size, four (8.3%) were microadenomas, and 42 (87.5%) were macroadenomas, including two (4.2%) giant ones. 20 adenomas were invasive. In two patients, the adenoma co-secreted prolactin. Hypopituitarism, that is, deficiency in at least one pituitary axis, was diagnosed in 23 (47.9%) of the cases. All patients were undergoing replacement therapy for the affected axes, except for GH. Regarding treatment, 46 underwent surgery, followed by radiotherapy in 9 cases. In 22 cases (45.8%), patients were using a somatostatin analog or pegvisomant at the last visit.

The follow-up was on average 113 months (range 3-252 months). At the last consultation, 46 patients returned with IGF-1 results. The number of patients with IGF-1 ≤ 1.0 × ULN considered “cured” was 18 (39.2%), biochemically controlled with medical therapy was 6 (13%), and with uncontrolled disease was 22 (47.8%). Considering IGF-1 ≤ 1.2 × ULN, 20 (43.5%) patients were considered “cured”, 6 (13%) were controlled with medical therapy, and 20 (43.5%) had uncontrolled disease. Considering an upper reference limit for IGF-1 of 1.2 × ULN, 18 patients were cured with surgery alone, 2 with surgery and radiotherapy, 3 achieved biochemical control with surgery and medical therapy, 2 with surgery, radiotherapy, and medical therapy, totaling 25 cases (54.3%). Table 1 summarizes the demographic and clinical characteristics of the study population.

**Table 1:** Demographic and clinical profile of the study population

Variables	*n* (%)
Age at presentation (years)	51 ± 11.4
Gender – Male	23 (47.9%)
BMI at presentation (kg/m2)	28.8 ± 4
Macroadenoma at presentation	42 (87.5%)
Tumoral invasion	20 (41.7%)
GH at presentation(mcg/L)	9.3 [IQR 3.7; 33.7]
IGF-1 at presentation(mcg/L)	736 [IQR 578; 857]
Hyperprolactinemia, *n* = 44*	12 (25%)
Hypopituitarism	
Some hypopituitarism**	23 (47.9%)
Hypoadrenocorticism	5 (10.4%)
Hypothyroidism	15 (31.3%)
Hypogonadism, *n* = 44*	12 (25%)
GH at last visit (mcg/L)	0.7 [IQR 0.2; 3.4]
IGF-1 at last visit, *n* = 46 (mcg/L)*	231 [IQR 154; 285]
Therapy (*n* = 48)	
Only medical therapy	2 (4.2%)
Only surgery	23 (47.9%)
Surgery and medical therapy (nova categoria) 14 (29,2%) Surgery and radiotherapy	3 (6.2%)
Surgery, radiotherapy and medical therapy	6 (12.5%)

Mean ± SD, except for GH and IGF-1, where IQR is listed. BMI, body mass index; GH, growth hormone; IGF-1, insulin-like growth factor-1; SSA, somatostatin analogs. * Variables with missing values ** Deficiency in at least one pituitary axis

### Joint assessment

Spontaneous joint complaints were reported by 23/43 patients (53.4%), 12 male and 11 female. On the specific questionnaire, 41 patients (85.4%) reported one or more complaints. The median number of joint complaints was 3. Table 2 details the complaint locations and the number of patients who reported them. Table 2 presents the distribution of signs/symptoms and referred pain locations.

**Table 2:** Signal/symptom and referred location (*n*=48)

Signal/Symptom and Referred Location	*n*	%
Pain	36	75.0
Upper Limb	12	33.3
Lower Limb	7	19.4
Spine	2	5.6
Multiple	15	41.7
Crepitation	24	50.0
Upper Limb	3	12.5
Lower Limb	12	50.0
Multiple	9	37.5
Stiffness	23	47.9
Upper Limb	6	26.1
Lower Limb	14	60.9
Spine	3	13.0
Edema	22	45.8
Upper Limb	6	27.3
Lower Limb	8	36.4
Spine	1	4.5
Multiple	7	31.8
Impaired Mobility	21	43.8
Upper Limb	9	42.9
Lower Limb	11	52.4
Multiple	1	4.8
Inability of Extension	19	39.6
Upper Limb	10	52.6
Lower Limb	6	31.6
Spine	1	5.3
Multiple	2	10.5
Instability	8	16.7
Upper Limb	1	12.5
Lower Limb	6	75.0
Multiple	1	12.5

**Fig. 1:**
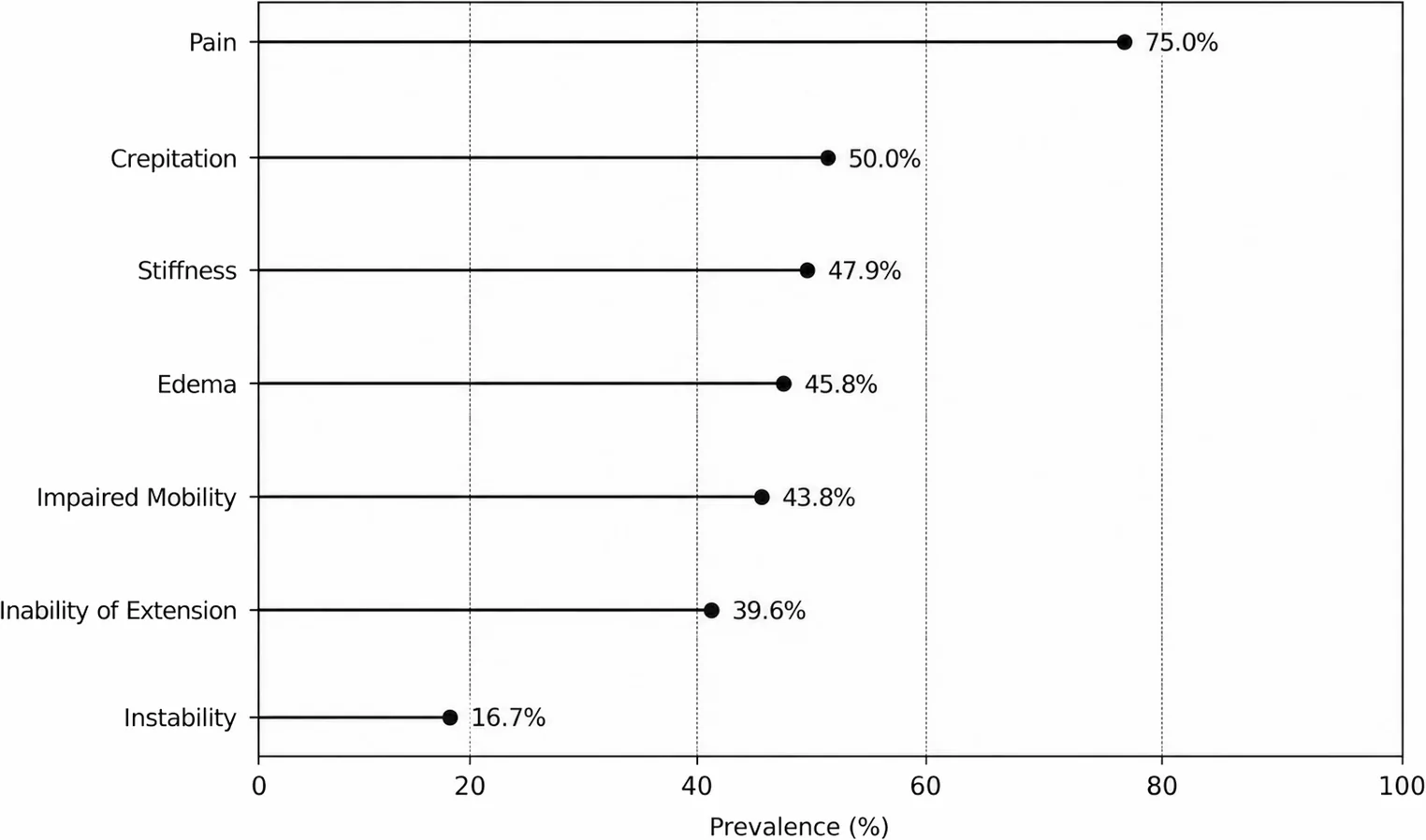
Frequency of each sign or symptom (*n*=48)

Figure 1 shows the frequency of complaints.

**Fig. 2:**
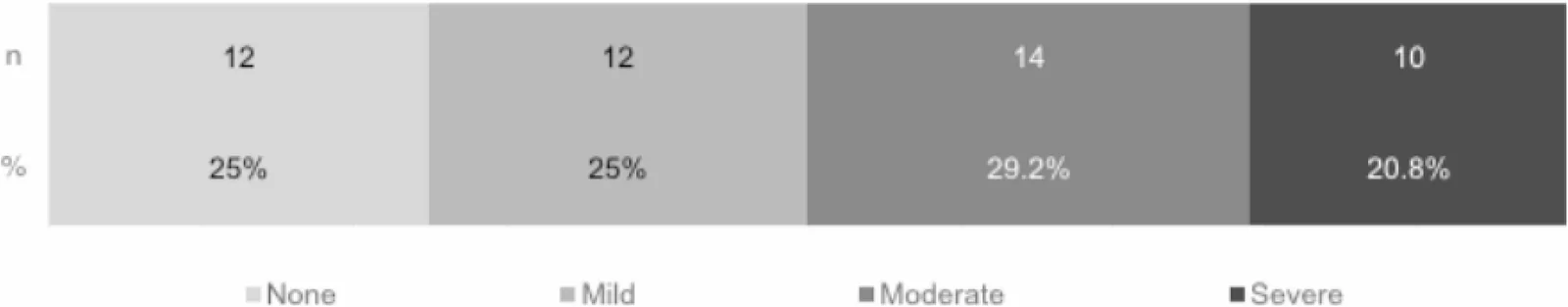
Degree of joint limitation. *QuickDASH questionary; *n* = 48

According to the QuickDASH questionnaire, 12 patients (25%) reported no upper-limb functional limitations during the previous week. A total of 36 patients (75%) exhibited some degree of upper-limb functional limitation, including mild limitation in 12 patients, moderate limitation in 14, and severe limitation in 10. Figure 2 shows the degree of joint mobility limitation.

Radiological examination of the upper limbs (hands and shoulders) was performed on 27 patients, 21 of whom (77.7%) showed some joint abnormalities. In 8 cases (29.7%), no radiological changes were observed in the hands (scores of zero or 1), while 70.3% of patients presented significant radiological changes in one or more joints. In the first fingers, 13 cases showed some articular alteration (score > 2); in 16 cases, the sum of the metacarpophalangeal and proximal and distal interphalangeal joints was > 2. In 16 cases (59.3%), patients presented significant radiological changes in one or both shoulder joints. Glenohumeral osteoarthritis was diagnosed in 13 cases (48.1%) and acromioclavicular osteoarthritis in 10 cases (37%).

Positive correlations were observed between age at presentation and radiographic change score (*r* = 0.636, *p* < 0.001).

Regarding employability, 26/43 (60.5%) patients were employed, with 23 in permanent paid jobs and 3 in temporary jobs. The remaining 17 patients (39.5%) were classified as unemployed. Among the employed, the median age was 55 years, and 4 (15.3%) patients were elderly; among the unemployed, the median age was 63 years, and 8 (47%) patients were elderly (*p* = 0.003). Among those employed, 11 (42.3%) had joint complaints, and 11 (42.3%) had uncontrolled disease. Among the unemployed, these numbers were 11 (64.7%) and 4 (23.5%), respectively, with no significant difference between the groups.

## Discussion

The sample’s demographic profile showed an age range consistent with the literature [14] and an equal distribution between sexes, consistent with previous studies that have not identified significant differences in acromegaly prevalence between men and women [15–18]. Similarly, most cases had a macroadenoma, as previously reported [14]. Prolactin-secreting tumors, strictly defined as prolactin levels greater than 200 ng/mL, were observed in 4.2% of cases. The literature reports varying prevalence of co-secretors depending on the criteria used. Ho et al. cite 14% co-secretors, using immunohistochemical criteria [19]. Petrossians and colleagues, evaluating more than 3,000 patients with acromegaly, reported co-secretion in 10% of cases, while in patients who underwent surgery, mixed GH/PRL staining was described in 26.3% of tumors [20]. A recent study of 178 patients who underwent surgery to remove somatotroph adenomas reported that 47% of patients used medication as adjuvant therapy [21]. In our cohort, 45.8% of patients were using post-surgical drug treatment at the last evaluation.

GH plays an important role in protein-mediated bone formation and is involved in cell proliferation and differentiation, factors that contribute to bone growth [22], thereby contributing to joint involvement in acromegaly. According to Kellgren, among the earliest reports of bone/joint effects of acromegaly are Marie’s 1886 descriptions of rheumatic crises with pain in the limbs and back region, then considered prominent [23]. This was followed by descriptions of painful, crepitant, and enlarged joints, and numerous other reports linking acromegaly to bone outcomes [23]. The pathophysiological findings of osteoarthritis differ from those in acromegaly. While individuals without excessive IGF-1 production typically exhibit joint space narrowing due to cartilage loss, most patients with acromegaly present with joint space widening, reflecting cartilage hypertrophy, often accompanied by severe osteophytosis characterized by bony growths at the joint margins [13, 24].

Joint manifestations typically appear around 10 years after disease onset [5]. Given the similar diagnostic delay observed in acromegaly, arthropathy may be the first manifestation of the disease.

Regarding pain symptoms, patients with acromegaly have higher joint pain scores in all joints except the elbow [25]. Pain is usually most prevalent in the knee and hip regions, whereas involvement of the hands, back, shoulders, feet, and neck is less frequent [6, 26]. Dons et al. reported arthralgia in 76% of cases, and Wassenaar et al. reported it in 72% [5, 10]. In the review by Claessen et al., 72% of patients reported pain or swelling in one or more joints, with the hands generating the most complaints (81%) [27]. Kyriakakis et al. reported shoulder pain in 38.6% of patients in their series [26]. In the current study, 53.4% of patients reported spontaneous joint complaints affecting multiple joints, while in the systematic interview, 85.4% presented one or more complaints. The main complaint reported was pain, more frequent in the upper limbs than in the lower limbs or spine.

Patients with acromegaly had more functional impairment [25]. Fatti et al. reported a significantly higher prevalence and severity of motor disability in acromegalics compared to the general population [28]. Yuen et al. demonstrated a 10- to 15-fold increase in medical costs and direct and indirect costs in patients with acromegaly compared to individuals without the disease [29].

Radiological changes were found in 77.7% of 27 cases submitted to radiological evaluation in the present study, with hand involvement observed in 70.3% of cases. Claessen and colleagues found radiographic osteoarthritis features in 84% of knees, 72% of hips, and 90% of hands [30]. In a review on the subject, and specifically in relation to the hands, osteoarthritis was observed in 43 to 61% of cases, depending on the affected joint [27]. According to Pelsma et al., radiographic osteoarthritis of the hands was the most frequent finding, occurring in 84.3% of cases [9].

Radiological evaluation of the shoulders in individuals with acromegaly is not frequently reported in the literature. Glenohumeral osteoarthritis was the most common shoulder abnormality in this study, affecting 48.1% of evaluated patients. In a cohort of individuals without acromegaly who attended a private clinic for shoulder complaints (*n* = 1001), glenohumeral osteoarthritis was diagnosed in 2.2% of the sample, predominantly after age 50, peaking between 60 and 69 years, and more common in women [31]. In patients with acromegaly, Fatti et al. observed shoulder arthropathy in 34% of patients and Kropf et al. in 35% [28, 32]. Pelsma et al. observed radiographic glenohumeral osteoarthritis in 21 patients (41.2%), a number slightly lower than that observed here [9]. For these authors, age and higher pre-treatment IGF-1 levels were associated with an increased risk of radiographic glenohumeral lesions. Other factors associated with radiographic progression of osteoarthritis include female sex, especially women over 50 years of age [31, 32], increased body mass index [27, 28], and prior radiotherapy [33]. We did not find a correlation between arthropathy and BMI. As previously mentioned, our negative correlation between IGF-1 levels at presentation and radiographic score may be attributable to sample size.

Interestingly, patients currently receiving pharmacological treatment compared to patients cured by surgery and/or radiotherapy presented a higher risk for radiographic glenohumeral osteoarthritis [9]. In an editorial, Romijn previously noted that medically well-controlled patients show greater radiographic progression than surgically cured patients [34].

Previous reports found no association between arthropathy and serum GH and IGF-I levels or disease duration until diagnosis [32]. The assessment of the potential association between GH/IGF-1 levels and the severity of arthropathy must be interpreted with caution, given that these variables are sometimes evaluated at patient presentation and at other times at different stages of disease progression—whether regarding the arthropathy or the acromegaly itself—and are frequently considered in isolation from the functional impact of the arthropathy. Studies have shown little promise regarding the reversibility of arthropathies, even after remission of acromegaly [24, 30]. Acromegalic arthropathy showed significant radiographic progression over 9.1 years of follow-up in patients in remission, whereas clinical progression was observed less frequently [13].

Despite therapeutic advances, acromegaly remains associated with considerable symptom burden and impaired health-related quality of life [35]. Beyond the visible burden of acromegaly, which includes the consequences of GH hypersecretion, tumor mass effects, and pituitary hormone deficiencies, patients may experience several additional challenges. These include persistent quality-of-life impairment; negative effects on partnerships, interpersonal relationships, and self-esteem; treatment-related burdens and adverse effects; limitations in daily activities due to cognitive impairment and disability; and financial difficulties related to reduced work capacity or occupational disability [36]. As acromegaly is most often diagnosed during a patient’s most productive years, and morbidity and functional disability can hinder employment, leading to reduced income and social interaction, it is important to consider the effects of acromegaly on patients’ employability. The percentage of unemployed patients observed here was five times higher than Brazil’s unemployment rate in the 4th quarter of 2025 (5.1%) [12]. Using a work productivity scale, Fatti et al. evaluated 80 patients with acromegaly who were actively employed and found a 7.89% loss of work time, a 1.96% impairment in work productivity, and a 2.98% impairment in regular daily activities [28]. Another study found that 16% of patients reported work absences attributable to treatment burden [36]. Among biochemically controlled patients, 92% reported symptom-related interference with daily life and 87% reported interference with work activities [37]. In Yuen et al.‘s assessment of costs and absenteeism among employees with acromegaly, 19.1% of employees with acromegaly (*n* = 47) had arthritis, compared with 3.7% of employees without acromegaly (*n* = 940). In the present study, unemployed patients were significantly older, and we found no significant difference between employed and unemployed patients in joint complaints or disease control.

This study has some limitations, particularly regarding sample size, owing to its single-center nature and the rarity of the disease. Other limitations include that a single reader performed the radiographic assessment and that the study lacked a control group. However, its prospective design and robust follow-up in a well-characterized cohort are important strengths. The study could be strengthened by including temporality variables, such as disease duration prior to diagnosis, duration of active disease, time since biochemical control was achieved, and current age.

The current study suggests a high frequency of joint complaints in patients with acromegaly, supported by radiological findings. The functional limitations detected here, although predominantly mild to moderate, can affect patients’ daily lives. This scenario underscores the need for systematic evaluation of joint involvement in patients with acromegaly, both at presentation and during follow-up, to enable timely therapeutic interventions that limit the progression of joint damage and alleviate pain.

## Data Availability

No datasets were generated or analysed during the current study.
